# Machine Learning Prediction of Quantum Yields and Wavelengths of Aggregation-Induced Emission Molecules

**DOI:** 10.3390/ma17071664

**Published:** 2024-04-04

**Authors:** Hele Bi, Jiale Jiang, Junzhao Chen, Xiaojun Kuang, Jinxiao Zhang

**Affiliations:** College of Chemistry and Bioengineering, Guilin University of Technology, Guilin 541006, China; bihele@163.com (H.B.);

**Keywords:** aggregation-induced emission, machine learning, quantum yield, wavelength

## Abstract

The aggregation-induced emission (AIE) effect exhibits a significant influence on the development of luminescent materials and has made remarkable progress over the past decades. The advancement of high-performance AIE materials requires fast and accurate predictions of their photophysical properties, which is impeded by the inherent limitations of quantum chemical calculations. In this work, we present an accurate machine learning approach for the fast predictions of quantum yields and wavelengths to screen out AIE molecules. A database of about 563 organic luminescent molecules with quantum yields and wavelengths in the monomeric/aggregated states was established. Individual/combined molecular fingerprints were selected and compared elaborately to attain appropriate molecular descriptors. Different machine learning algorithms combined with favorable molecular fingerprints were further screened to achieve more accurate prediction models. The simulation results indicate that combined molecular fingerprints yield more accurate predictions in the aggregated states, and random forest and gradient boosting regression algorithms show the best predictions in quantum yields and wavelengths, respectively. Given the successful applications of machine learning in quantum yields and wavelengths, it is reasonable to anticipate that machine learning can serve as a complementary strategy to traditional experimental/theoretical methods in the investigation of aggregation-induced luminescent molecules to facilitate the discovery of luminescent materials.

## 1. Introduction

The development of efficient organic luminescent materials is crucial for high-performance organic light-emitting diodes [1,2,3], biological probes [4,5], and chemical sensors [6,7,8]. Organic luminescent materials have attracted extensive attention from researchers in various fields due to their intriguing biocompatibility, structural diversity, and ease of property tuning [9,10,11]. However, traditional organic luminescent materials usually suffer from luminescence quenching at high concentrations or in the aggregated states, which severely limits their practical applications [12,13,14]. Fortunately, Tang et al. coined the term “aggregation-induced emission (AIE)” and paved a practical way to enhance the emission efficiency of molecules in the aggregated states [15,16]. Since then, luminogens with AIE property (AIEgens) have served as essential luminescent materials, with widespread application potential in optoelectronic devices [17,18], biological imaging [19], and energy conversion [20]. Luminescence quantum yields (*Φ*) and maximum absorption/emission wavelengths (*λ_abs_*, *λ_em_*) are two important optical parameters of AIEgens in the applications of AIEgens, especially material development, mechanistic study, and high-tech applications [21,22,23]. Rational design of potential AIEgens with desired wavelengths and quantum yields is the key to achieving favorable luminescent materials.

Traditional experimental methods often adopt a trial-and-error approach, which demands high resources and is time-consuming to obtain high-performance AIE molecules, especially when the chemical compositions and structures are complex and diverse [24,25,26]. Quantum chemical methods such as density functional theory (DFT) can predict the wavelengths and quantum yields of molecules without chemical synthesis, but they fail to obtain AIE molecules in bulk [21,27]. Computer-aided chemistry has taken many forms in recent decades. The use of machine learning (ML) has proliferated in order to drastically reduce design and experimental effort [28,29,30]. Therefore, there is an urgent need to bypass traditional tedious experimental exploration and theoretical calculation processes and combine emerging ML methods with luminescent chemistry to achieve rapid and accurate predictions of luminescent properties from their molecular structures [31,32,33].

ML is gaining increasing popularity in scientific research and has been extensively utilized in various areas, including luminescent materials, organic synthesis, and drug design [34]. For nonexperts lacking an understanding of the underlying physical and chemical mechanisms between molecular structures and properties [35], ML can help them directly predict a wide range of physical and chemical properties based on molecular features extracted from molecular structures [36]. For researchers who already possess some foundational knowledge, ML can offer supplementary insights to assist them in developing molecules with expected properties efficiently. In the luminescent domain, Ju et al. used structural and solvent descriptors to construct accurate ML models for predicting the photophysical properties *(λ_abs_*, *λ_em_*, and *Φ*) of distinct organic fluorescent molecules [37]. Shao et al. developed a new ML model based on deep neural networks for the accurate prediction of the maximum absorption wavelengths for a carefully prepared database of solvated small molecular fluorophores [38]. Senanayake et al. proposed three classification and regression ML machines for predicting the emission color and wavelengths of carbon dots. The best models achieved up to 94% accuracy for emission color and a minimum mean average error of 25.8 nm for wavelength, facilitating the design of carbon dots with targeted optical properties [39]. Mahato et al. optimized a series of ML models to predict the physical properties of organic dyes, and the derived R^2^ values for absorption and emission wavelengths that were 0.7% and 0.4% larger, respectively, than those recently reported by the gradient boosted regression (GBR) models [40].

In the field of AIE materials, the incorporation of ML has greatly facilitated materials screening and discovery, as well as the characterization of the structural–optical properties [41]. Qiu et al. proposed an efficient ML scheme based on quantum mechanics to classify AIE and aggregation-induced quenching (ACQ) properties of diverse triphenylamine derivatives, relying on their luminescent moieties [42]. Xu et al. developed an ensemble strategy to predict the optical properties of organic molecules in the aggregated states, wherein multiple prediction methods were designed, compared, and combined to achieve an optimized multimodal approach [43]. Zhang et al. reported a multimodal molecular descriptors strategy to extract the structure–property relationships of AIEgens and predict the absorption and emission wavelengths peaks of the molecules, and three newly predicted AIEgens with the desired absorption and emission wavelengths were successfully applied to cellular fluorescence imaging and deep penetration imaging [44]. Given the successful applications of ML methods in luminescent materials, it is reasonable to speculate that ML holds significant potential in predicting wavelengths and quantum yields, both of which are two important factors of AIEgens [37].

In this work, we employed ML methods to predict the quantum yields and absorption/emission wavelengths of 563 organic molecules in the monomeric/aggregated states, collected from literature reports spanning several years. Molecular fingerprints were chosen as ML inputs, and favorable molecular fingerprints were selected by comparing 13 different individual molecular fingerprints and various combined molecular fingerprints. Afterwards, different ML algorithms were applied to the selected favorable molecular fingerprints and further compared to obtain the best ML models. The predicted quantum yields and absorption/emission wavelengths are in good agreement with reference values. The predicted accuracy of the optimal ML models was further confirmed with DFT calculations for four newly designed AIE molecules. Therefore, our ML approach is expected to provide new ideas and methods for the development and application of aggregation-induced luminescent materials.

## 2. Materials and Methods

**Figure 1:**
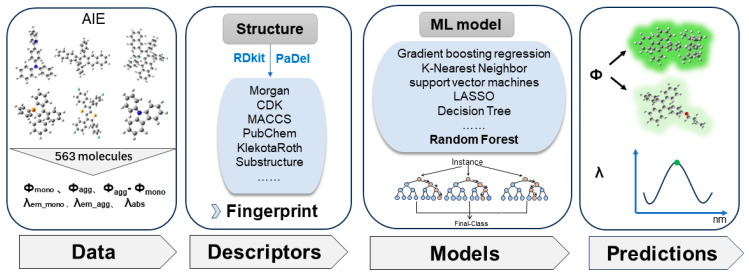
Workflow of machine learning (ML) approach in predicting the luminescence properties (quantum yield *Φ* and wavelength *λ*) of luminogens with aggregation-induced emission property (AIEgens) in the monomeric/aggregated states. The workflow consists of four steps: collecting molecular structures and their corresponding *Φ*/*λ* data; extracting molecular descriptors from molecular structures; optimizing ML models by performing different ML algorithms on different molecular descriptors; predicting *Φ*/*λ* with ML models for new molecules.

In this study, we applied a ML approach to predict the quantum yields and absorption/emission wavelengths of 563 organic molecules in both monomeric and aggregated states. The methodology involved four key steps, as illustrated in Figure 1: data collection, extraction of molecular descriptors, training of ML models, and ML predictions. We carefully constructed a database of the photophysical properties of about 563 organic luminescent compounds in both the monomeric and aggregated forms, collected from the research literature on AIE over the years. The emission wavelengths and quantum yields of molecules in both the original states (monomer, mostly in tetrahydrofuran solution) and the aggregated states (mostly in tetrahydrofuran solution with a water content of more than 90% or in solid state) were collected because the photophysical properties of luminescent molecules are usually influenced by their aggregation states due to the AIE and ACQ effects. Each organic luminescent molecule in the database includes six photophysical properties: maximum absorption wavelengths (*λ_abs_*), maximum emission wavelengths in the monomeric and aggregated states (*λ_em_mono_*, *λ_em_agg_*), quantum yields in the monomeric and aggregated states (*Φ_mono_*, *Φ_agg_*), and their difference (*Φ_agg_-Φ_mono_*). The database was randomly divided into three subsets for benchmarking: the training, validation, and test sets, with respective ratios of about 65%, 15%, and 20%. The training set was utilized for the ML training to learn and establish relationships between ML inputs and outputs. The validation set was employed for tuning hyperparameters and preventing overfitting to the training set during the ML training process. The test set was used for evaluating the final performance of the trained ML models [45]. The dataset for ML training (the training and validation sets) contained about 463 samples, and the test set outside the training group (out-of-sample dataset) included about 100 samples.

Afterwards, the molecular structures were converted to molecular descriptors as ML inputs. Molecular descriptors are the mathematical representations of compounds, which can capture diverse parts of the structural information of molecules. Molecular fingerprint is a typical type of molecular descriptor where structural features are converted to either binary bits in a bit vector or counts in a count vector [46,47]. Molecular fingerprints hold richer structural and physicochemical information compared to some simple molecular descriptors [48,49,50]. Thirteen molecular fingerprints, which have proved their performance in predicting luminescent properties in previous reports, were selected as ML input candidates: MACCS (MA), Morgan, AtomPairs2D, PubChem (P), Substructure (S), Estate (E), CDK (CDK), CDKextended (CDKex), SubstructureCount (Sc), AtomPairs2DCount, CDKgraphonly, KlekotaRoth (K), and KlekotaRothCount (Kc) fingerprints. The 13 molecular fingerprints of the 5,10-diphenylphenazine (DPhPZ) molecule were used as examples and were listed in Table S1 to enlighten the forms of molecular fingerprints. The preferred fingerprints were combined to create combined molecular fingerprints to further enhance the efficiency and accuracy of ML. All of the molecular fingerprints were generated using RDKit and PaDEL-Descriptor packages with SMILES strings as inputs. SMILES strings can be exported (Figure S1) after creating 2D molecular structures in ChemDraw [51,52].

Subsequently, ML training was carried out to achieve optimal ML models [53,54]. The selection of ML algorithms is crucial for the accuracy of ML predictions. For quantum yield predictions (*Φ_mono_*, *Φ_agg_*, *Φ_agg_-Φ_mono_*), five typical classification ML algorithms were chosen: random forest (RF), decision tree (DT), naive Bayes (NB), K-nearest neighbor (KNN), and support vector machine (SVM). To develop more appropriate binary classifiers, we used the median of the experimental quantum yields (5%) as the threshold to divide the database into categories of high-efficiency luminescence (>5%) and low-efficiency luminescence (<5%). The evaluation of the algorithms’ performance included metrics such as the receiver operating characteristic curve (ROC), area under the curve (AUC), accuracy rate (ACC), and F1-score (F1). For wavelength predictions (*λ_abs_*, *λ_em_mono_*, *λ_em_agg_*), four regression ML algorithms were selected: RF, KNN, GBR, and least absolute shrinkage and selection operator (LASSO) regression algorithms, which were all adopted in the prediction of wavelengths in recent reports [55,56]. Pearson correlation coefficient (*r*), mean relative error (MRE), and mean absolute error (MAE) were used to evaluate the algorithms’ performances. After the ML training, we saved the ML models with the best performance in the validation sets for six photophysical properties. The ML predictions were carried out on the test set to further evaluate the performances of optimal ML models. Finally, four new AIE molecules were designed and their quantum yields were predicted with ML models, which were confirmed with DFT calculations. All the ML training procedures were carried out using the Python language in the Jupyter Notebook editor of the Anaconda platform [57]. The open-source toolkit scikit-learn was used to process data (including fingerprint conversion, train–test splitting), import, and tweak various ML classification and regression algorithms for ML tasks. The DFT calculations were carried out in Gaussian 16 [58]. More details can be found in the Supplementary Materials.

## 3. Result and Discussions

### 3.1. Prediction of Quantum Yields in the Aggregated and Monomeric States

**Figure 2:**
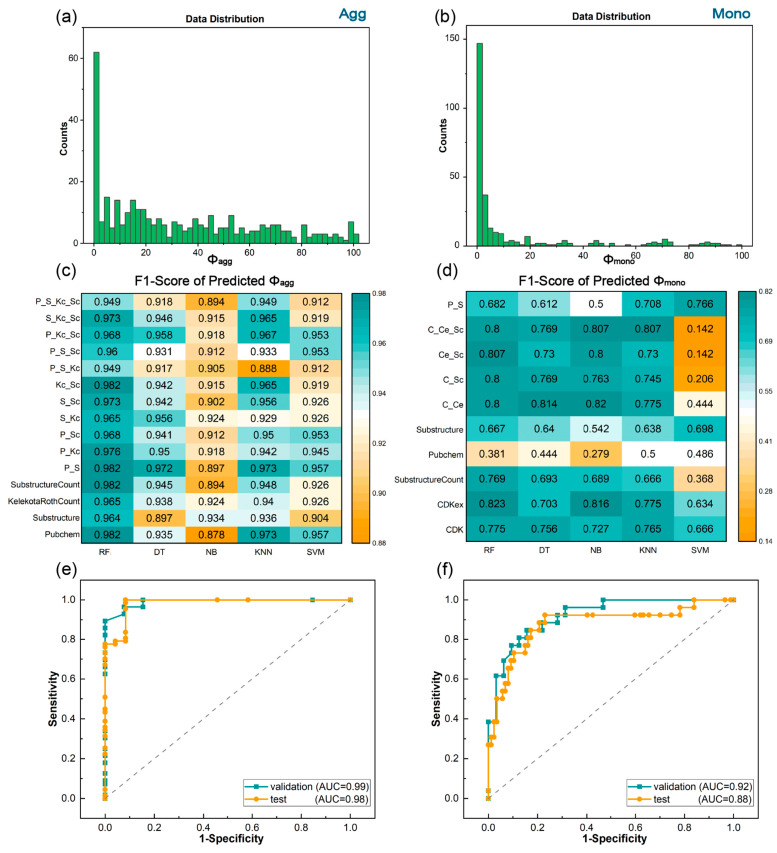
The data distributions and ML results of *Φ_agg_* and *Φ_mono_*_._ Data distribution of (**a**) *Φ_ag_*_g_ and (**b**) *Φ_mono_*. Heat map of F1-scores predicted with different fingerprints and ML classification algorithms of (**c**) *Φ_agg_* and (**d**) *Φ_mono_*. ROC curves of validation set predicted in ML training process and test set predicted with the optimal ML trained models for (**e**) *Φ_agg_* and (**f**) *Φ_mono_*.

The RF algorithm not only demonstrates superior performance in handling high-dimensional features during the prediction of molecular luminescence properties but also exhibits robustness to outliers [59,60,61,62]. Therefore, the RF algorithm was chosen in combination with 13 individual molecular fingerprints candidates for ML training to gain a general understanding of their prediction effects, as shown in Table 1. The quantum yields serve as a crucial factor in evaluating the luminescence efficiency of organic molecular materials. Thus, we firstly carried out the ML training for the quantum yields in the aggregated states. The data distribution of quantum yields (Figure 2a) showed a peak near zero and a relatively average distribution in most regions because most of the molecules exhibit low or even no luminescence, and high-performance luminescent molecules are rare. This highlights the urgency of molecular design and selection to achieve highly efficient luminescent organic molecules. Four individual fingerprints showed relatively high performance in the predictions of *Φ_agg_*: PubChem, Substructure, KelekotaRothCount, and SubstructureCount fingerprints (Table 1). The AUC values of the four fingerprints were all above 0.90, and their ACC and F1-scores were both above 0.93 (Table S3).

Combined fingerprints were constructed with four selected preferred fingerprints to enhance the accuracy and efficiency of structural representations because an individual fingerprint may not be able to fully represent the structural information of a molecule under certain conditions [63]. Figure 2c lists 11 combined fingerprints consisting of 2–4 kinds of individual fingerprints, all displaying superior performance in ML tasks. The first column of Figure 2c exhibits the F1-scores of ML training results of the RF classifier algorithm based on 11 combined fingerprints. It is obvious that P_S and Kc_Sc fingerprints exhibited F1-scores of 0.98 (Figure 2c), with AUC values reaching 0.99 and ACC up to 0.97 (Table S4).

Subsequently, 4 individual and 11 combined fingerprints were trained under different ML algorithms to identify optimal ML models because the selection of an appropriate ML algorithm will influence the prediction accuracy of molecular luminescence properties. The ML training results revealed that the RF algorithm showed the best performance in predicting *Φ_agg_*. Characterized as a versatile ensemble learning methodology, the RF algorithm demonstrates the capability to handle mixed data within its framework. This proficiency arises from the inherent nature of its tree growth and splitting process, which naturally accommodates both continuous and categorical data [64,65]. Consequently, the RF algorithm exhibited commendable stability when applied to our dataset. In contrast, the ACCs of DT, NB, and SVM were observed to be moderate. A combined fingerprint, P_S, exhibited the best result compared with other fingerprints across different ML algorithms. Therefore, in the aggregated states, the RF algorithm in conjunction with the P_S fingerprint (RF/P_S) model exhibited the best prediction results, and its ROC curve was depicted in Figure 2e, reaching an AUC of 0.99, an ACC of 0.97 (Table S4), and an F1-score of 0.98 (Figure 2c).

For the prediction of quantum yields in the monomeric state (*Φ_mono_*), we employed the optimal molecular descriptor, P_S fingerprint, identified from the prediction of *Φ_agg_,* in combination with the same five binary classification algorithms. Unfortunately, the predictive performance of the P_S fingerprint proved unsatisfactory across the five ML algorithms (Figure 2d). Therefore, similar to the prediction process of *Φ_agg_*, 13 individual molecular fingerprints candidates were combined with RF for ML training to screen out preferable fingerprints. It can be seen from Table 1 that three fingerprints—CDK, CDKex, and SubstructureCount—achieved AUC and ACC both above 0.84. The three fingerprints were combined to construct combined fingerprints and were severed as ML inputs for five ML algorithms to acquire optimal ML models. Similar to the aggregated state, RF algorithm was superior to other algorithms in the monomeric state, with KNN ranking second as shown in Figure 2d. RF/CDKex yielded the best ML models, with AUC of 0.92, ACC of 0.84 (Table S5), and F1-score of 0.82 (Figure 2d). Therefore, it can be inferred that the RF binary classifier model with suitable molecular fingerprints can provide reasonable predictions for quantum yields, and the optimal methods are RF/P_S for *Φ_agg_* and RF/CDKex for *Φ_mono_*.

In the ML training process, the validation set acted as a checkpoint for refining the ML models, independent of the test set, helping to improve the model’s performance on unseen data. The optimal ML models were saved after ML training. Subsequently, the test set was used for evaluating the final performance of the well-trained ML models to new data. The optimal ML models were employed in the test set, which comprised approximately 100 samples outside the training set, to predict the photophysical properties, and their prediction results were compared with the reference values. Figure 2e,f presents ROC curves of the validation set and the test set of optimal models under the aggregated and monomeric states, respectively. It is evident that the AUC for the validation sets was notably high in ML training. The AUC value for the aggregated state in the test set was up to 0.98, suggesting the high robustness of the optimal model, and can be used to discriminate aggregate-induced organic materials with strong luminescence (*Φ* > 5%), thereby facilitating the screening of AIE candidates. Although the prediction performance of quantum yields under the monomeric state was slightly inferior, its AUC value in the test set still reached 0.88 (Figure 2f), indicating a satisfactory capability to predict quantum yields in the monomeric state. The successful prediction of *Φ_agg_* and *Φ_mono_* in the test set verified the prediction accuracy for new data.

In order to further evaluate the prediction accuracy of the optimal ML models, we designed four new organic molecules (Figure S3a) and compared their ML-predicted quantum yields with DFT calculated results. The ML predictions revealed that the four molecules displayed weak emission in the monomeric states, but high quantum yields after aggregation (Table S10). A high quantum yield can be achieved with a fast intersystem crossing rate (*k_ISC_*) between the singlet and triplet excited states of molecules. Therefore, we used the calculated *k_ISC_* to evaluate the quantum yields predicted by the ML models. A large *k_ISC_*, *k_ISC_*∝|⟨S_m_|H_SO_|T_n_⟩|^2^/(ΔE_S-T_)^2^ [66,67], can be realized by enhancing the spin-orbit couplings (SOC, ⟨S_m_|H_SO_|T_n_⟩) and reducing the energy gap (ΔE_S-T_) between the singlet excited state and the triplet excited state. As shown in Figure S3b, the excited energy levels underwent energy splitting in the process of aggregation due to excitonic coupling, resulting in more energy channels for ISC, thereby reducing ΔE_S-T_. The SOCs of aggregates were comparable to those of monomers (Tables S11 and S12). Subsequently, the *k_ISC_* for the dominant channel S_1_-T_n_ increased after aggregation (Figure S3c). Additionally, the high-lying excited states also displayed significant *k_ISC_*, which further facilitates the overall *k_ISC_* in the aggregated states. Therefore, the DFT calculated results confirmed the high luminescent properties of the four newly designed AIE molecules, as predicted by the optimal ML models, indicating that the optimal model can assist in designing high-performance new AIE molecules.

### 3.2. Prediction of the Quantum Yield Difference between the Aggregated and Monomeric States

**Figure 3:**
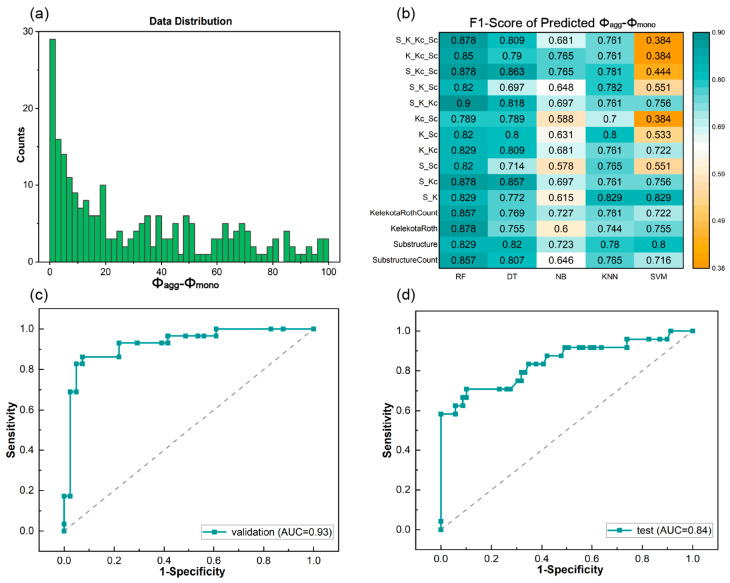
The data distributions and ML results of *Φ_agg_-Φ_mono_*. (**a**) Data distribution. (**b**) Heat map of F1-scores predicted with different fingerprints and ML classification algorithms. The receiver operating characteristic curve (ROC) curves of (**c**) validation set predicted in ML training process and (**d**) test set predicted with the optimal ML trained model.

ML training was also performed for the difference in quantum yields before and after aggregation (*Φ_agg_-Φ_mono_*) because the relative value can reduce system error due to the different experimental conditions in the collected literature. The relative value (*Φ_agg_-Φ_mono_*) can serve as a measure of the change in luminescence intensity before and after molecular aggregation. Figure 3a illustrates the distribution of *Φ_agg_-Φ_mono_*, where the median value (25%) was chosen as the threshold for the ML model.

Table 1 lists the ML results of 13 individual molecular fingerprints with the RF algorithm. The top four molecular fingerprints, Substructure, SubstructureCount, KelekotaRoth, and KelekotaRothCount, with AUC > 0.90, ACC > 0.85, and F1-scores > 0.82 (Table S3), were adopted to generate 11 combined fingerprints for five ML training algorithms. Similar to the predictions of absolute values (*Φ_agg_* and *Φ_mono_*), the RF with combined fingerprints (RF/S_K_Kc) model revealed the highest accuracy in our database. Its F1-score reached 0.90 (Figure 3b), AUC reached 0.93, and ACC reached 0.91 (Table S6). The prediction result of the RF/S_K_Kc model in the test set exhibited AUC of 0.84 (Figure 3d) and ACC of 0.86 (Table S7), verifying its favorable prediction ability. The RF algorithm in combination with combined fingerprints demonstrated commendable accuracy and robustness in predicting quantum yields.

### 3.3. Prediction of Emission Wavelengths and Absorption Wavelengths

**Figure 4:**
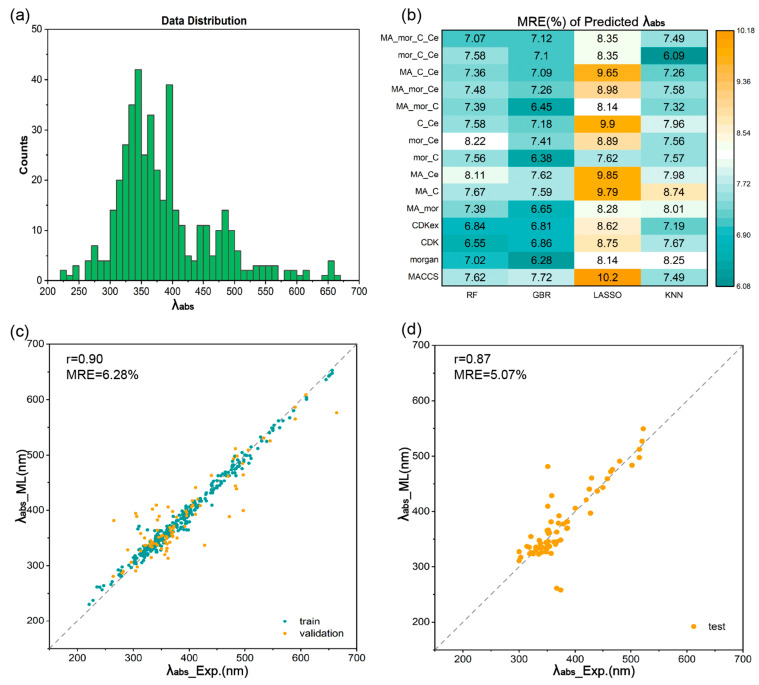
The data distributions and ML results of *λ_abs_*. (**a**) Data distribution of *λ_abs_*. (**b**) Heat map of mean relative error (MRE) of *λ_abs_* predicted with different fingerprints and ML regression algorithms. Regression curves of (**c**) training set and validation set predicted in ML training process and (**d**) test set predicted with the optimal ML trained model for *λ_abs_*.

The prediction of the absorption and emission wavelengths (*λ_abs_* and *λ_em_*) of organic luminescent molecules across a spectrum of wavelengths holds significant importance for their photochemical applications, such as spectral analysis, laser processing, photocatalysis, and photosensitive materials [68,69]. Figure 4a shows the data distribution of *λ_abs_* within the range of 300–700 nm collected from the literature, and the data exhibits a normal distribution, which validates the reliability of our data. Similar to the process employed for predicting quantum yields, we firstly used the RF algorithm to filter out individual fingerprints with commendable performance, yielding the following results: MACCS, Morgan, CDK, and CDKex (Table 1). Subsequently, the four individual fingerprints were combined to attain combined fingerprints for further ML training with four ML regression algorithms. The performance metrics analysis in Figure 4b reveal that the MRE range for both RF and GBR was within 8.22%, making them two preferable regression algorithms for *λ_abs_*. The Morgan fingerprint under GBR algorithm exhibited the smallest MRE at 6.28% compared to other fingerprints and ML methods. The regression curve of the validation set of optimal model (GBR/Morgan) is shown in Figure 4c, with an *r* value of 0.90, achieving the expected effect. The verification of the prediction accuracy of the optimal model was performed in the test set, and the final result in the test set yielded an *r* of 0.87 and an MRE of 5.07% (Figure 4d), demonstrating the substantial robustness of the optimal ML model in predicting absorption wavelengths.

**Figure 5:**
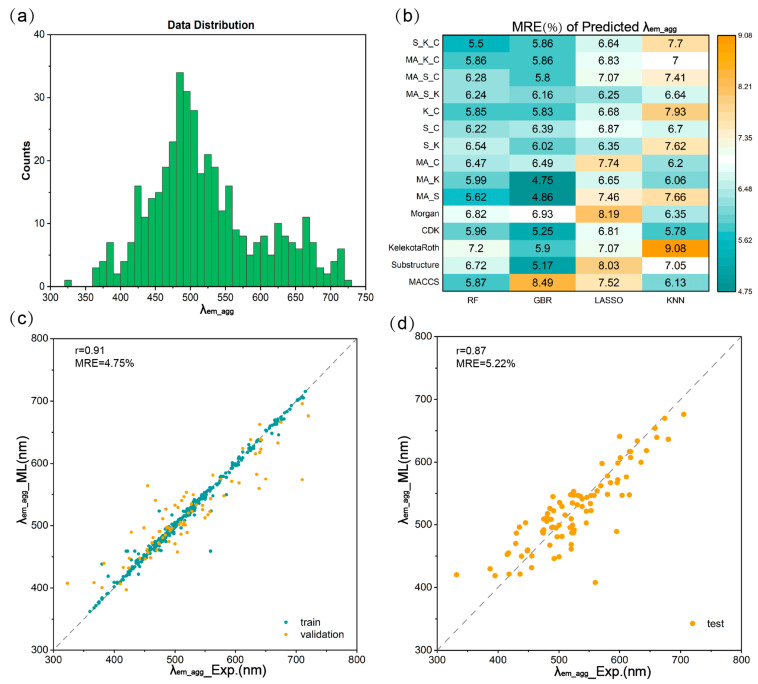
The data distributions and ML results of *λ_em_agg_*. (**a**) Data distribution of *λ_em_agg_*. (**b**) Heat map of MRE of *λ_em_agg_* predicted with different fingerprints and ML regression algorithms. Regression curves of (**c**) training set and validation set predicted in ML training process and (**d**) test set predicted with the optimal ML trained model for *λ_em_agg_*.

We extended our study to explore the emission wavelengths of molecules in both the aggregated and monomeric states (*λ_em_agg_* and *λ_em_mono_*). Figure 5a presents the data distribution for *λ_em_agg_*, fitting well to a normal distribution. The optimal fingerprints for *λ_abs_* (the Morgan fingerprint) were adopted and compared with 13 individual molecular fingerprints for ML training under RF algorithm. Four fingerprints, MACCS (*r* = 0.84, MRE = 5.87%), Substructure (*r* = 0.82, MRE = 6.72%), SubstructureCount (*r* = 0.83, MRE = 6.91%) and KelekotaRoth *(r* = 0.83, MRE = 7.20%), demonstrated superior performance compared with Morgan fingerprint (*r* = 0.76, MRE = 6.82%), as revealed in Table 1. To evaluate the effects of combined fingerprints, the selected four fingerprints were combined into 11 combined fingerprints and served as ML inputs for ML training with four regression algorithms. The results revealed that MA_K combined fingerprints trained using GBR regression exhibited the lowest MRE value of 4.75%, as indicated in Figure 5b. The regression curve for the optimal model (GBR/MA_K) illustrated a favorable *r* of 0.91 and an MRE of 4.75% (Figure 5c).

GBR/MA_K was adopted for further prediction of emission wavelengths in the monomeric state (*λ_em_mono_*), which also demonstrated satisfactory results. To validate whether the GBR/MA_K method remains the optimal ML model for predicting *λ_em_mono_*, we compared the results with screened individual molecular fingerprints (Table 1) and combined fingerprints based on screened fingerprints under four different ML algorithms. It was found that the MA_K/GBR method held superior results when compared to other methods, with an MRE of 6.27% and an *r* of 0.92 (Table S9, Figure S2). In summary, the GBR/MA_K model was the optimal model for predicting emission wavelengths under both the aggregated and monomeric states. The prediction results were close to experimental data, with *r*-values of 0.91 and 0.92 for *λ_em_agg_* and *λ_em_mono_*, respectively, and MRE of 4.75% for *λ_em_agg_* and 6.27% for *λ_em_mono_* (Figure 5c and Figure S2).

The well-trained models were employed to predict the emission wavelengths in both the aggregated and monomeric states for the test set. As illustrated in Figure 5d, the regression curve derived from the aggregated state in the test set yielded a commendable *r* of 0.87, accompanied by an MRE of 5.22%. Similarly, in the monomeric state, the outcomes from the test set yielded an *r* of 0.88 and an MRE of 4.31% (Figure S2d). These observed errors fall within an acceptable range, demonstrating the model’s robustness and precision in predicting emission wavelengths and, thereby, affirming its utility in practical applications.

The successful prediction of quantum yields and wavelengths of AIE molecules by our optimal ML models is beneficial for researchers interested in AIE molecules. For those new to AIE research, our optimal ML models enable the prediction of quantum yields and wavelengths for a large number of organic molecules, facilitating the screening of potential AIE molecules without requiring an in-depth understanding of structure–property relationships. Experienced researchers in the luminescent domain can use their chemical expertise and understanding of structure–property relationships in AIE molecules to design new structures with potentially high quantum yields by including propeller-like or rotor features, such as tetraphenylethylene (TPE) and triphenylamine (TPA), to restrict molecular motions. They can also design new structures of AIE molecules with potentially long emission wavelengths by introducing electron donor and acceptor groups into a π-conjugation system, extending the π-conjugation degree and reducing the bandgap in AIE molecules. Subsequently, they can employ our optimal ML models to predict quantum yields and wavelengths and further identify new structures with expected AIE properties.

## 4. Conclusions

In this work, a series of ML trainings were carried out to achieve the fast and accurate prediction of quantum yields and absorption/emission wavelengths. Optical properties data of about 563 organic luminescent molecules in both the aggregated and monomeric states were collected from the literature reported in recent years. Molecule structures were then converted into a variety of machine-readable individual and combined molecular fingerprints. Different ML algorithms were chosen for ML training, using different individual/combined molecule fingerprints as ML inputs to screen out the optimal fingerprints and ML algorithms. Rapid and robust predictions were achieved for six optical properties: *Φ_mono_*, *Φ_agg_*, *Φ_agg_-Φ_mono_*, *λ_abs_*, *λ_em_mono_*, and *λ_em_agg_*. (1) For quantum yield predictions, we used a classification model to distinguish strong and weak quantum yields of luminescent materials. The best model for predicting quantum yields in the aggregated state in the validation set was found to be RF/P_S, which achieved an AUC of 0.99, ACC of 0.97, and F1-score of 0.98. The model also demonstrated favorable prediction accuracy and robustness in the test set (AUC = 0.98, ACC = 0.97). The best model for quantum yields in the monomeric state was RF/CDKex, with an AUC of 0.92 and ACC of 0.84 in the validation set, and yielding a good robustness results in the test set (AUC = 0.88, ACC = 0.85). The prediction accuracy and robustness of the optimal ML models were verified by DFT calculations for four newly designed AIE molecules. The high accuracy of the quantum yields prediction suggest the high effectiveness of our ML model in differentiating high and low quantum yield intensities in both the monomeric and aggregated states. This may prove to be useful in identifying organic luminescent molecules with strong quantum yields. (2) For wavelength predictions, we established optimal regression models for predicting both absorption and emission wavelengths in the monomeric and aggregated states. For the aggregated state, the optimal model for predicting emission wavelengths was GBR/MA_K, with an *r* of 0.91 and an MRE of 4.75% in the validation set. This model maintained its effectiveness in the test set, achieving an *r* of 0.87 and an MRE of 5.22%. Additionally, four newly designed AIE molecules were predicted using the optimal ML models and successfully verified with DFT calculations, suggesting the prediction accuracy of the optimal ML models and their potential for designing new AIE molecules.

Our results indicate that the utilization of combined fingerprints in the aggregated state can lead to better accuracy in predicting quantum yields compared to individual fingerprints. In addition, the RF classification algorithm was proven to be the best ML method for predicting quantum yields, and the GBR regression method was optimal for predicting wavelengths. The ML models developed in this study can facilitate the screening of organic molecules with desired photophysical properties, thus reducing traditional experimental/computational resource and time costs. Furthermore, these models can aid in the design of new AIEgens, thereby promoting the development of high-performance organic luminescent materials.

## Figures and Tables

**Table 1:** Evaluation results of 13 individual fingerprints in different properties under RF algorithm.

Descriptors	*Φ_agg_*		*Φ_mono_*		*Φ_agg_-Φ_mono_*		*λ_abs_*		*λ_em_agg_*		*λ_em_mono_*	
	AUC	ACC	AUC	ACC	AUC	ACC	r	MRE/%	r	MRE/%	r	MRE/%
MACCS	0.73	0.9	0.87	0.77	0.88	0.81	0.81	7.62	0.84	5.87	0.86	7.15
Morgan	0.82	0.82	0.86	0.84	0.83	0.81	0.85	7.02	0.76	6.82	0.83	7.56
Atomp	0.74	0.86	0.71	0.87	0.82	0.79	0.70	8.38	0.77	7.57	0.70	10.0
Pubchem	0.92	0.97	0.60	0.56	0.89	0.80	0.75	8.59	0.80	7.21	0.83	9.80
Substructure	0.90	0.94	0.81	0.72	0.94	0.85	0.73	7.34	0.82	6.72	0.83	8.25
Estate	0.88	0.91	0.81	0.81	0.86	0.78	0.69	7.58	0.79	7.15	0.84	7.81
CDK	0.82	0.93	0.91	0.87	0.84	0.83	0.82	6.55	0.81	5.96	0.82	7.96
CDKex	0.82	0.93	0.92	0.84	0.83	0.80	0.80	6.84	0.81	6.98	0.78	8.79
SubstructureCount	0.92	0.93	0.87	0.84	0.90	0.89	0.75	9.00	0.83	6.91	0.82	8.03
Atompair2DCount	0.79	0.93	0.84	0.72	0.84	0.80	0.74	8.18	0.80	8.10	0.79	8.89
CDKgraphonly	0.82	0.91	0.85	0.71	0.82	0.73	0.72	9.50	0.80	6.76	0.73	10.2
KlekotaRoth	0.88	0.95	0.86	0.79	0.94	0.90	0.72	8.66	0.83	7.20	0.84	8.37
KlekotaRothCount	0.90	0.94	0.82	0.78	0.93	0.87	0.76	7.91	0.82	7.00	0.84	7.95

## Data Availability

The data used in this work are available in https://github.com/bihele/AIE_database, accessed on 27 March 2024.

## Supplementary Materials

The following supporting information can be downloaded at: https://www.mdpi.com/article/10.3390/ma17071664/s1, Table S1: Thirteen molecular fingerprints of DPhPZ molecule. Table S2: ML algorithms employed in this work. Table S3: F1-scores of Φ predicted with RF algorithm under different individual fingerprints. Table S4: AUC and ACC of *Φ_agg_* predicted with different ML algorithms under different combined fingerprints. Table S5: AUC and ACC of *Φ_mono_* predicted with different ML algorithms under different fingerprints. Table S6: AUC and ACC of *Φ_agg_-Φ_mono_* predicted with different ML algorithms under different combined fingerprints. Table S7: ML-predicted results of the test set with the optimal models for *Φ_agg_*, *Φ_mono_* and *Φ_agg_*-*Φ_mono_*. Table S8: MRE and *r* of *λ_abs_* predicted with different ML algorithms under different combined fingerprints. Table S9: MRE and *r* of *λ_em_mono_* predicted with different ML algorithms under different combined fingerprints. Table S10. The predicted quantum yield results for the four newly designed molecules under the ML optimal models. Table S11. Calculated SOC for the S_1_-T_n_ channel of the four molecules in monomeric states. Table S12. Calculated SOC for the S_1_-T_n_ channel of the four molecules in the aggregated states. Figure S1. The 2D structure and SMILES string of the DPhPZ molecule. Figure S2: The data distributions and ML results of *λ_em_mono_.* Figure S3. The four newly designed AIE molecules and their DFT calculation results.
