# Supplementary material for: Machine Learning Prediction of Quantum Yields and Wavelengths of Aggregation-Induced Emission Molecules
Source: Materials (Basel). 2024 Apr 4;17(7):1664. doi: 10.3390/ma17071664 (PMC11012915; doi:10.3390/ma17071664)
Supplement: Supplementary file 1 [file materials-17-01664-s001.zip › materials-2914327-supplementary.pdf]

materials MDPI

Supplementary materials

Machine Learning Prediction of Quantum Yields and Wavelengths of Aggregation-Induced Emission Molecules

Hele Bi, Jiale Jiang, Junzhao Chen, Xiaojun Kuang and Jinxiao Zhang *

College of Chemistry and Bioengineering, Guilin University of Technology, Guilin 541006, China; bihele@163.com (H.B.); jialejiangchuzhou@163.com (J.J.); comezhao@yeah.net (J.C.); kuangxj@glut.edu.cn (X.K.)
* Correspondence: jxzh@glut.edu.cn

1. Materials and Methods

1.1 Molecular Descriptors

In a typical machine learning (ML) training process, molecular structures are firstly transformed into molecular descriptors and serve as inputs for ML training. Molecular descriptors are the mathematical representations of compounds. They encode a wide variety of molecular information, including diverse aspects of geometry, shape, pharmacophores, or atomic properties of molecules. Molecular fingerprint is a specific type of molecular descriptor where structural features are converted to either binary bits in a bit vector or counts in a count vector [1,2]. All molecular fingerprints are molecular descriptors, but not all molecular descriptors are fingerprints. Molecular descriptors provide a broader range of information about a molecule, whereas molecular fingerprints are more focused on the structural representation. Therefore, molecular fingerprints contain richer structural and physicochemical information compared to some simple molecular descriptors, which were chosen as ML inputs in this study [3–5]. The 2D structures of the molecules were created and transferred to SMILES strings in ChemDraw. Subsequently, these SMILES strings were converted to Morgan fingerprints using the RDkit package, and to MACCS (MA), AtomPairs2D, PubChem (P), Substructure (S), Estate (E), CDK (CDK), CDKextended (CDKex), SubstructureCount (Sc), AtomPairs2DCount, CDKgraphonly, KlekotaRoth (K), and KlekotaRothCount (Kc) fingerprints using the PaDEL-Descriptor package [6,7]. In order to illustrate the forms of molecular descriptors, we used the DPhPZ molecule as an example, and presented its molecular 2D structure (Figure S1), SMILES string (Figure S1), and 13 molecular fingerprints (Table S1）.

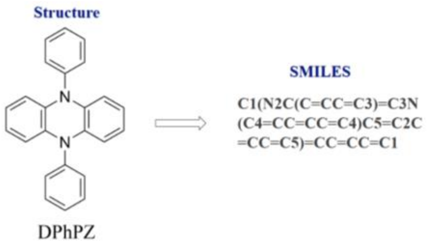

Figure S1. The 2D structure and SMILES string of the DPhPZ molecule.

Table S1. Thirteen molecular fingerprints of the DPhPZ molecule.

Descriptors	Fingerprints	Bits
MACCS	000000000000000000000000000……000010100111010	166
Morgan	000000000000000000000000010……000000000000000	1024
Atomp	110000000000000000000000000……000000000000000	780
Pubchem	111000000111101100000000000……000000000000000	880
Substructure	000000000000000000000010000……000100000100001	307
Estate	000000000001000010000000000……000000000000000	79
CDK	000100000000010000100000000……000100100000000	1024
CDKex	000000010000000000000000011……111111100000000	1024
SubstructureCount	000000000000000000000020000……000000002000026	307
Atompairs2DCount	246000000000000000000000000……000000000000000	780
CDKgraphonly	000000000000010000001000001……100000000000000	1024
KlekotaRoth	000000000000000000000000000……000000000000000	4860
KlekotaRothCount	000000000000000000000000000……000000000000000	4860

1.2 Database and Train–Test Splitting

The database, a collection of approximately 563 molecules with AIE properties, was derived from previously published works and included 6 luminescent properties (Φ_{mono}, Φ_{agg}, Φ_{agg}-Φ_{mono}, λ_{abs} ,λ_{em_mono}, λ_{em_agg}). When performing ML training on each photophysical property, a very small number of samples with missing values were removed from the molecular dataset. For the purpose of benchmarking, the dataset was randomly divided into three subsets: the training, validation, and test sets, adhering to proportions of 65%, 15%, and 20%, respectively. The training set and validation set were utilized in the ML training process, which contained about 463 samples. The training set is used to build the ML model and helps fit the model's parameters, thus allowing the ML algorithm to learn the relationship between inputs and outputs. The validation set is used to tune the hyperparameters of the model and check for overfitting. It acts as a checkpoint for refining the model, independent of the test set, helping to improve the model's performance on unseen data. The test set, comprising approximately 100 samples outside the training data, was utilized to assess the prediction accuracy of the well-trained ML models to new data.

Table S2. ML algorithms employed in this work.

Type	Python code for importing required libraries	Algorithms
Classifier	from sklearn.ensemble import RandomForestClassifier	RF
	from sklearn.svm import SVR	SVR
	from sklearn.tree import DecisionTreeClassifier	DT
	from sklearn.naive_bayes import MultinomialNB	NB
	from sklearn.neighbors import KNeighborsClassifier	KNN
Regressor	from sklearn.ensemble import RandomForestRegressor	RF
	from sklearn.ensemble import GradientBoostingRegressor	GBR
	from sklearn import neighbors	KNN
	from sklearn.linear_model import Lasso	LASSO

1.3 Machine Learning Metrics

MRE (mean relative error) for n samples is given by the following:

$$MRE = \frac{1}{n}\sum_{i=1}^{n}\frac{\left|y_{pred}^{(i)} - y_{true}^{(i)}\right|}{y_{true}^{(i)}} \in [0, +\infty]$$

MAE (mean absolute error) for n samples is given by the following:

$$MAE = \frac{1}{n}\sum_{i=1}^{n}\left|y_{true}^{(i)} - y_{pred}^{(i)}\right| \in [0, +\infty]$$

r (Pearson's correlation coefficient) for n samples is given by the following:

$$r = \frac{\sum_{i=1}^{n}(y_{true}^{(i)} - \overline{y_{true}})(y_{pred}^{(i)} - \overline{y_{pred}})}{\sqrt{\sum_{j=1}^{n}(y_{true}^{(i)} - \overline{y_{true}})^2 \sum_{k=1}^{n}(y_{pred}^{(k)} - \overline{y_{pred}})^2}} \in [-1, 1]$$

ACC (accuracy): ACC is commonly utilized as a standard metric for evaluating accuracy. The conventional format for evaluating accuracy is the confusion matrix, also known as the error matrix, which intuitively characterizes the predictive performance of the model. The matrix is structured into four quadrants:

True positive (TP): The sample is predicted to be positive and is actually positive.

True negative (TN): The sample is predicted to be negative and is actually negative.

False positive (FP): The sample is predicted to be positive but is actually negative.

False negative (FN): The sample is predicted to be negative but is actually positive.

The ACC is calculated as follows:

$$ACC = \frac{TP + TN}{TP + TN + FN + FP}$$

ACC serves as the primary evaluation index, particularly effective for binary classifiers. However, when the class is unbalanced, ACC may exhibit bias towards the majority class and has little effect on the evaluation of minority classes. Therefore, two additional comprehensive indicators, F1-score and AUC, are employed.

F1 (F1-score): A higher F1-score indicates superior results. The F1-score for n samples is given by the following:

$$F1 = \frac{2 \times \frac{TP}{TP + FP} \times \frac{TP}{TP + FN}}{\frac{TP}{TP + FP} + \frac{TP}{TP + FN}}$$

To explain the area under the curve, it is necessary to introduce the concepts of true positive rate (TPR) and false positive rate (FPR). Both indicators are based on the conditional probability of true labels, unaffected by the true distribution of labels.

$$TPR = \frac{TP}{TP + FN} \qquad FPR = \frac{TP}{TN + FP}$$

AUC (area under the curve): The receiver operating characteristic curve (ROC) utilizes FPR as the abscissa and TPR as the ordinate, generating curves with varying thresholds. AUC is the area under the ROC curve. Basically, a larger AUC or closer proximity of the curve to the upper-left corner (TPR = 1, FPR = 0) signifies superior model performance.

1.4 DFT and TDDFT Calculation Details

All the density functional theory (DFT) and time-dependent DFT (TDDFT) calculations were carried out using the Gaussian 16 package [8]. B3LYP and CAM-B3LYP hybrid exchange-correlation functionals were employed for the ground state geometry optimizations and the excited state TDDFT simulations, respectively. A Gaussian basis set of 6-311g(d,p) was used to describe molecular structures. The Grimme' D3 dispersion correction term with Becke–Johnson damping parameters (DFT-D3-BJ) was used to consider van der Waals dispersion. The spin–orbital couplings (SOC) were calculated with the PySOC program [9]. An SCRF solvent model with water as solvent was employed for all the calculations.

2. Computational Results

Table S3. F1-scores of *Φ* predicted with RF algorithm under different individual fingerprints.

Descriptors	Φ_{agg}	Φ_{mono}	Φ_{agg}-Φ_{mono}
MACCS	0.918	0.723	0.780
Morgan	0.834	0.823	0.769
Atomp	0.938	0.667	0.790
Pubchem	0.982	0.381	0.808
Substructure	0.964	0.667	0.829
Estate	0.954	0.784	0.760
CDK	0.929	0.775	0.800
CDKex	0.912	0.823	0.780
SubstructureCount	0.982	0.769	0.857
Atompairs2DCount	0.940	0.714	0.800
CDKgraphonly	0.945	0.701	0.711
KlekotaRoth	0.937	0.739	0.878
KlekotaRothCount	0.965	0.785	0.857

Table S4. AUC and ACC of Φ_{agg} predicted with different ML algorithms under different combined fingerprints.

Φ_{agg}	Metrics	RF	DT	NB	KNN	SVM
P_S	AUC	0.99	0.94	0.92	0.98	0.98
	ACC	0.97	0.95	0.84	0.95	0.92
P_Kc	AUC	0.94	0.81	0.73	0.80	0.76
	ACC	0.95	0.91	0.85	0.89	0.89
P_Sc	AUC	0.97	0.83	0.90	0.81	0.95
	ACC	0.94	0.92	0.86	0.88	0.86
S_Kc	AUC	0.98	0.86	0.87	0.93	0.95
	ACC	0.94	0.89	0.85	0.91	0.91
S_Sc	AUC	0.99	0.92	0.87	0.91	0.93
	ACC	0.95	0.91	0.84	0.92	0.86
Kc_Sc	AUC	0.99	0.92	0.80	0.97	0.95
	ACC	0.97	0.91	0.85	0.94	0.85
P_S_Kc	AUC	0.97	0.78	0.85	0.75	0.80
	ACC	0.91	0.86	0.84	0.81	0.84
P_S_Sc	AUC	0.97	0.84	0.91	0.80	0.95
	ACC	0.92	0.88	0.85	0.88	0.91
P_Kc_Sc	AUC	0.98	0.85	0.86	0.83	0.95
	ACC	0.94	0.92	0.85	0.94	0.91
S_Kc_Sc	AUC	0.99	0.88	0.83	0.97	0.96
	ACC	0.95	0.91	0.85	0.94	0.85
P_S_Kc_Sc	AUC	0.97	0.82	0.86	0.89	0.90
	ACC	0.91	0.86	0.82	0.91	0.84

Table S5. AUC and ACC of Φ_{mono} predicted with different ML algorithms under different fingerprints.

Φ_{mono}	Metrics	RF	DT	NB	KNN	SVM
CDK	AUC	0.91	0.83	0.84	0.86	0.87
	ACC	0.87	0.84	0.84	0.81	0.75
CDKex	AUC	0.92	0.72	0.86	0.87	0.87
	ACC	0.84	0.72	0.84	0.81	0.74
Substruc-	AUC	0.87	0.73	0.71	0.72	0.70
ture_Count	ACC	0.84	0.74	0.68	0.72	0.58
C_Ce	AUC	0.92	0.83	0.85	0.87	0.87
	ACC	0.82	0.82	0.84	0.81	0.65
C_Sc	AUC	0.92	0.79	0.83	0.81	0.80
	ACC	0.82	0.79	0.77	0.77	0.60
Ce_Sc	AUC	0.91	0.76	0.83	0.81	0.80
	ACC	0.82	0.75	0.81	0.75	0.58
C_Ce_Sc	AUC	0.92	0.79	0.84	0.86	0.80
	ACC	0.82	0.79	0.82	0.82	0.58

Table S6. AUC and ACC of Φ_{agg}-Φ_{mono} predicted with different ML algorithms under different combined fingerprints.

Φ_{agg}-Φ_{mono}	Metrics	RF	DT	NB	KNN	SVM
S_K	AUC	0.93	0.81	0.76	0.89	0.88
	ACC	0.85	0.78	0.68	0.85	0.85
S_Kc	AUC	0.93	0.88	0.78	0.86	0.87
	ACC	0.89	0.87	0.72	0.78	0.80
S_Sc	AUC	0.88	0.75	0.74	0.83	0.77
	ACC	0.85	0.74	0.65	0.76	0.72
K_Kc	AUC	0.93	0.83	0.78	0.86	0.85
	ACC	0.85	0.82	0.70	0.78	0.78
K_Sc	AUC	0.89	0.83	0.76	0.84	0.78
	ACC	0.85	0.82	0.70	0.80	0.70
Kc_Sc	AUC	0.86	0.82	0.72	0.80	0.86
	ACC	0.82	0.82	0.70	0.74	0.65
S_K_Kc	AUC	0.93	0.84	0.78	0.86	.88
	ACC	0.91	0.82	0.72	0.78	0.80
S_K_Sc	AUC	0.90	0.73	0.76	0.84	0.79
	ACC	0.85	0.72	0.72	0.78	0.72
S_Kc_Sc	AUC	0.92	0.88	0.79	0.83	0.84
	ACC	0.89	0.87	0.76	0.78	0.68
K_Kc_Sc	AUC	0.92	0.81	0.79	0.82	0.80
	ACC	0.87	0.80	0.76	0.78	0.65
S_K_Kc_Sc	AUC	0.93	0.83	0.78	0.81	0.84
	ACC	0.89	0.82	0.80	0.78	0.65

Table S7. ML-predicted results of the test set with the optimal models for Φ_{agg}, Φ_{mono} and Φ_{agg}-Φ_{mono}.

	Φ_{agg}	Φ_{mono}	Φ_{agg}-Φ_{mono}
ACC	0.97	0.85	0.86
F1_Score	0.98	0.70	0.69

Table S8. MRE and *r* of λ_{abs} predicted with different ML algorithms under different combined fingerprints.

λ_{abs}	Metrics	RF	GBR	LASSO	KNN
MA_mor	r	0.84	0.88	0.83	0.79
	MRE	7.39	6.65	8.28	8.01
MA_C	r	0.84	0.85	0.73	0.77
	MRE	7.67	7.59	9.79	8.74
MA_Ce	r	0.83	0.83	0.73	0.81
	MRE	8.11	7.62	9.85	7.98
mor_C	r	0.86	0.89	0.85	0.80
	MRE	7.56	6.38	7.62	7.57
mor_Ce	r	0.82	0.84	0.76	0.81
	MRE	8.22	7.41	8.89	7.56
C_Ce	r	0.81	0.82	0.71	0.84
	MRE	7.58	7.18	9.90	7.96
MA_mor_C	r	0.83	0.86	0.83	0.85
	MRE	7.39	6.45	8.14	7.32
MA_mor_Ce	r	0.82	0.82	0.81	0.81
	MRE	7.48	7.26	8.98	7.58
MA_C_Ce	r	0.83	0.84	0.71	0.78
	MRE	7.36	7.09	9.65	7.26
mor_C_Ce	r	0.81	0.85	0.82	0.85
	MRE	7.58	7.10	8.35	6.09
MA_mor_C_Ce	r	0.84	0.84	0.82	0.84
	MRE	7.07	7.12	8.35	7.49

Table S9. MRE and *r* of λ_{em_mono} predicted with different ML algorithms under different combined fingerprints.

λ_{em_mono}	Metrics	RF	GBR	LASSO	KNN
MA_Kc	r	0.90	0.87	0.83	0.85
	MRE	6.52	6.87	7.68	7.85
MA_K	r	0.91	0.92	0.86	0.87
	MRE	6.35	6.27	8.17	8.64
MA_S	r	0.90	0.87	0.82	0.86
	MRE	6.49	7.18	9.17	8.50
Kc_K	r	0.85	0.86	0.85	0.73
	MRE	7.99	8.35	8.35	10.5
Kc_S	r	0.90	0.87	0.83	0.81
	MRE	6.79	6.64	7.97	7.73
K_S	r	0.90	0.86	0.82	0.81
	MRE	6.64	6.60	8.57	9.98
MA_Kc_K	r	0.90	0.89	0.86	0.79
	MRE	7.65	7.28	7.67	10.30
MA_Kc_S	r	0.89	0.90	0.85	0.81
	MRE	7.65	6.78	8.12	9.10
MA_K_S	r	0.91	0.88	0.85	0.87
	MRE	6.31	6.37	8.14	8.40
Kc_K_S	r	0.91	0.86	0.84	0.81
	MRE	6.87	6.82	7.68	8.91

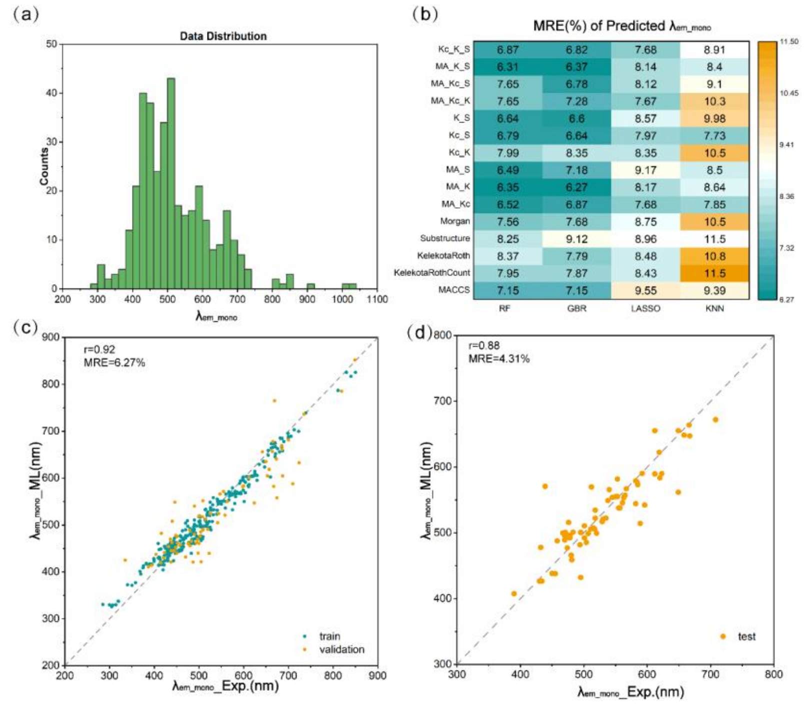

Figure S2. The data distributions and ML results of λ_{em_mono}. (a) Data distribution of λ_{em_mono}. (b) Heat map of MRE of λ_{em_mono} predicted with different fingerprints and ML classification algorithms. Regression curve of λ_{em_mono} of (c) training and validation sets predicted in ML training process and (d) test set predicted with the optimal ML trained models.

Table S10. The predicted quantum yield results for the four newly designed molecules under the ML optimal models.

Num	Φ_{mono}	Φ_{agg}
（1）	0	1
（2）	0	1
（3）	0	1
（4）	0	1

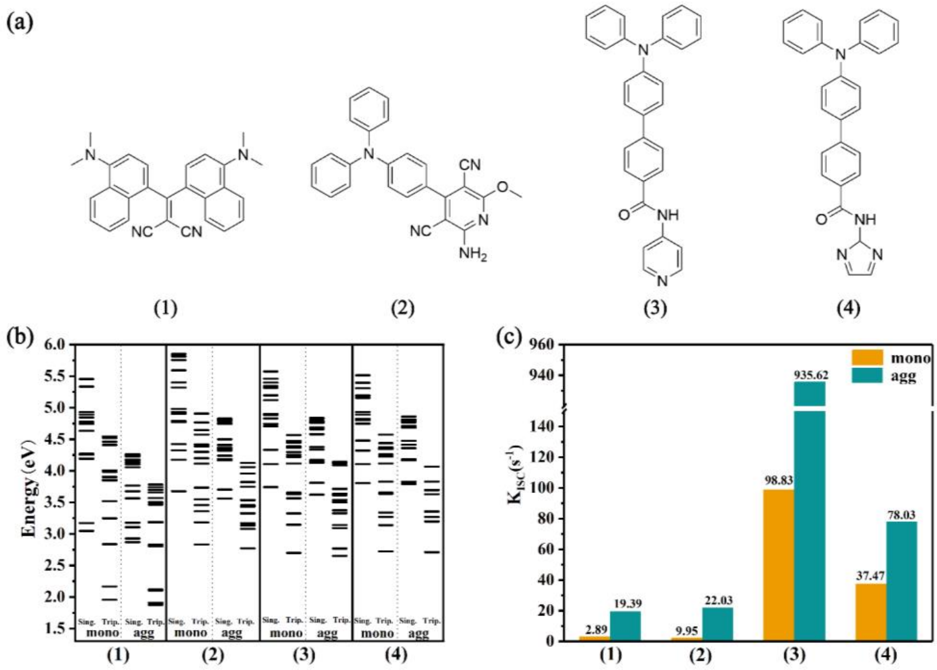

Figure S3. The four newly designed AIE molecules and their DFT calculation results. (a) Molecular structure of the four newly designed AIE molecules. (b) Calculated electronic energy levels of singlet and triplet excited states. (c) Calculated k_{ISC} for the ISC channel between the lowest singlet excited state and the adjacent triplet excited state.

Table S11. Calculated SOCs for the S_1-T_n channel of the four molecules in monomeric states.

SOC	No.	T_1	T_2	T_3	T_4	T_5	T_6
S_1	(1)	0.829	0.174	0.358			
	(2)	0.548	0.682	0.103	0.190	0.176	
	(3)	0.377	0.743	0.156	0.341	0.313	0.868
	(4)	0.291	0.798	0.071	0.214	0.305	0.934

Commented [EE1]: Please ensure intended meaning is retained
Response: We have revised the sentence in question to clarify its intended meaning, ensuring that the core message is conveyed more effectively and unambiguously.

Table S12. Calculated SOC for the S_1-T_n channel of the four molecules in aggregated states.

SOC	No.	T_1	T_2	T_3	T_4	T_5	T_6	T_7	T_8	T_9	T_{10}	T_{11}	T_{12}
S_1	(1)	0.601	0.318	0.159	0.255	0.393	0.176						
	(2)	0.533	0.109	0.154	0.735	0.093	0.108	0.188	0.049	0.125			
	(3)	0.372	0.026	0.138	0.762	0.269	0.048	0.398	0.236	0.303			
	(4)	0.168	0.168	0.600	0.285	0.058	0.053	0.117	0.210	0.236	0.192	0.369	0.821

References

1. Grisoni, F.; Ballabio, D.; Todeschini, R.; Consonni, V. Molecular descriptors for structure-activity applications: A hands-on approach. *Methods Mol Biol* **2018,** *1800*, 3-53.
2. Riniker, S.; Landrum, G.A. Similarity maps - a visualization strategy for molecular fingerprints and machine-learning methods. *J Cheminform* **2013,** *5*, 43.
3. Capecchi, A.; Probst, D.; Reymond, J.L. One molecular fingerprint to rule them all: Drugs, biomolecules, and the metabolome. *J Cheminform* **2020,** *12*, 43.
4. Yang, J.; Cai, Y.; Zhao, K.; Xie, H.;Chen, X. Concepts and applications of chemical fingerprint for hit and lead screening. *Drug Discov Today* **2022,** *27*, 103356.
5. Motiei, L.;Margulies, D. Molecules that generate fingerprints: A new class of fluorescent sensors for chemical biology, medical diagnosis, and cryptography. *Acc Chem Res* **2023,** *56*, 1803-1814.
6. Yap, C.W. Padel-descriptor: An open source software to calculate molecular descriptors and fingerprints. *J Comput Chem* **2011,** *32*, 1466-74.
7. Dong, J.; Cao, D.S.;Miao, H.Y.; Liu, S.; Deng, B.C.;Yun, Y.H.; Wang, N.N.; Lu, A.P.;Zeng, W.B..Chen, A.F. Chemdes: An integrated web-based platform for molecular descriptor and fingerprint computation. *J Cheminform* **2015,** *7*, 60.
8. Frisch, M.J.T., G. W.; Schlegel, H. B.; Scuseria, G. E.; Robb, M. A.; Cheeseman, J. R.; Scalmani, G.; Barone, V.; Petersson, G. A.; Nakatsuji, H.; Li, X.; Caricato, M.; Marenich, A. V.; Bloino, J.; Janesko, B. G.; Gomperts, R.; Mennucci, B.; Hratchian, H. P.; Ortiz, J. V.; Izmaylov, A. F.; Sonnenberg, J. L.; Williams-Young, D.; Ding, F.; Lipparini, F.; Egidi, F.; Goings, J.; Peng, B.; Petrone, A.; Henderson, T.; Ranasinghe, D.; Zakrzewski, V. G.; Gao, J.; Rega, N.; Zheng, G.; Liang, W.; Hada, M.; Ehara, M.; Toyota, K.; Fukuda, R.; Hasegawa, J.; Ishida, M.; Nakajima, T.; Honda, Y.; Kitao, O.; Nakai, H.; Vreven, T.; Throssell, K.; Montgomery, J. A., Jr.; Peralta, J. E.; Ogliaro, F.; Bearpark, M. J.; Heyd, J. J.; Brothers, E. N.; Kudin, K. N.; Staroverov, V. N.; Keith, T. A.; Kobayashi, R.; Normand, J.; Raghavachari, K.; Rendell, A. P.; Burant, J. C.; Iyengar, S. S.; Tomasi, J.; Cossi, M.; Millam, J. M.; Klene, M.; Adamo, C.; Cammi, R.; Ochterski, J. W.; Martin, R. L.; Morokuma, K.; Farkas, O.; Foresman, J. B.; Fox, D. J. Gaussian16 revision c.01. **2016**, Gaussian Inc. Wallingford CT.
9. Gao, X.;Bai, S.; Fazzi, D.;Niehaus, T.; Barbatti, M.; Thiel, W. Evaluation of spin-orbit couplings with linear-response time-dependent density functional methods. *J Chem Theory Comput* **2017,** *13*, 515-524.
